# Lost in Translation—How Transparency Can Improve Comparability and Reusability in Acoustic Bat Research

**DOI:** 10.1002/ece3.71883

**Published:** 2025-07-29

**Authors:** Jarno Asmus, Karl‐Heinz Frommolt, Mirjam Knörnschild

**Affiliations:** ^1^ Museum für Naturkunde Leibniz‐Institute for Evolution and Biodiversity Science Berlin Germany; ^2^ Evolutionary Ethology, Institute for Biology Humboldt‐Universität Zu Berlin Berlin Germany; ^3^ Deutsche Fledermauswarte e.V. Berlin Germany

**Keywords:** acoustic monitoring, activity metrics, bats, call identification, population data, standardization

## Abstract

Imagine solving a puzzle where half the pieces are missing: Acoustic bat monitoring, a key method for studying species distribution and population shifts, depends on purpose‐specific parameters and clear documentation. Without detailed information on these specific parameters, mainly the employed hard‐ and software, reproducibility and cross‐study comparability are not given. This comparability is imperative to address the gaps in bat population data, which have been described as one of the major threats to global bat biodiversity. We aimed to identify the parameters critical for the accurate interpretation and reproducibility of acoustic bat monitoring and assess how these parameters are communicated in existing studies. Using the PRISMA guidelines, we systematically reviewed studies that employed acoustic bat surveys or monitoring in European forests since 2008. Our review focused on three primary components: (1) recording devices and their settings, (2) call identification protocols, and (3) bat activity interpretation methods. Over 90% of the reviewed studies lacked basic methodological parameters. In all areas, we observed incomplete reporting of settings, unreproducible call identification protocols, and data interpretation methods that did not provide access to underlying data. We found a wide range of acoustic bat monitoring methods applied to diverse research questions. This variability underscores the fact that recommending a single approach is neither practical nor desirable. However, consistent reporting of equipment and methods is essential for improving transparency across studies. A standardized framework specifying key parameters for reporting would enhance comparability, support data reuse, and promote more robust bat monitoring and acoustic research. The lack of standardization does not reflect poorly on the researchers' competence or intent but emphasizes the need for a unified approach to bioacoustics methodology. We propose a standardized framework for reporting methodological parameters in acoustic bat monitoring to improve comparability, reproducibility, and transparency across studies.

## Graphical abstract

Acoustic monitoring is a key method for assessing bat populations and their ecological patterns, but the diversity of hardware and software requires transparent documentation to ensure reproducibility and comparability across studies. Our systematic review of European forest studies since 2008 found that over 90% lacked essential methodological details, particularly in recorder settings and configurations, call identification protocols, and data interpretation. To address this, we propose a standardized reporting framework to improve transparency, support data reuse, and enhance the reliability of acoustic bat research.

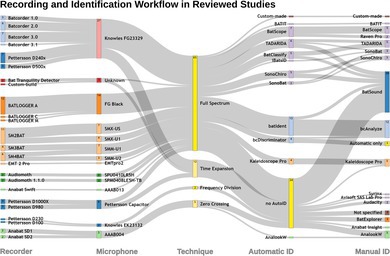

## 1 Introduction

Bats are cosmopolitan mammals, inhabiting diverse habitats ranging from forests and steppes to urban areas and deserts worldwide (Mickleburgh et al. 2002; Voigt and Kingston 2016). As the second most species‐rich taxonomic order of mammals, they play a vital role in ecosystems and provide essential ecosystem services (Ramírez‐Fráncel et al. 2022). These include pollination (Cordero‐Schmidt et al. 2021), seed dispersal (Villalobos‐Chaves and Rodríguez‐Herrera 2021), and insect pest suppression relevant to agriculture and forestry globally (Charbonnier et al. 2014; Russo, Bosso, and Ancillotto 2018; Maslo et al. 2022; Frank 2024). However, despite their ecological importance and their role as pest control in agriculture and forestry, bats are insufficiently protected and threatened by habitat loss and climate change (Festa et al. 2023). Their nocturnal and elusive lifestyle, as well as non‐standardized assessment methods, further complicate monitoring efforts, resulting in significant data gaps that hinder effective conservation (Russo et al. 2025). These gaps are further exacerbated by ongoing threats, such as climate change, land‐use changes, and habitat destruction (Frick et al. 2020; Festa et al. 2023). In the European Union, all bat species are protected under the EUROBATS international agreement, yet considerable knowledge gaps persist regarding European bat populations, including migration corridors and roosting sites (Browning et al. 2021). In addition, infrastructure projects like wind power plants continue to pose a significant threat to bat populations (Horn et al. 2008; Cryan et al. 2014).

Assessing bat populations and detecting potential community changes using bioacoustics is an effective, non‐invasive method (Box 1). It can provide a temporal snapshot of species presence or abundance (survey) or involve repeated sampling to track population trends and community dynamics (monitoring) (Frick 2013; Jones et al. 2013; Hoggatt et al. 2024). On a larger scale, bioacoustics enable the study of climate change‐driven shifts in bat populations (Amorim et al. 2014), alterations in activity patterns (Meramo et al. 2022; Kotila et al. 2023) and regional differences within species (Gillam and McCracken 2007). Locally, it can be applied to assess bat activity across different habitats. In forest environments, acoustic surveys and monitoring have been used to investigate the vertical stratification of bat species (Müller et al. 2013), responses of insectivorous bats to insect assemblages (Müller et al. 2012; Charbonnier et al. 2014; Blažek et al. 2021), and the influence of forest structure and composition on bat communities (Jung et al. 2012; Leidinger et al. 2021). Additionally, acoustic monitoring plays a key role in environmental impact assessments, such as evaluating the effects of infrastructure projects like wind power plants (Behr et al. 2023) or highways (Voigt and Kingston 2016).

BOX 1 **Basics of acoustic signal capture in bat monitoring.**

Acoustic surveys and monitoring of bats require a microphone and a recorder. The microphone detects bat calls, sound waves with specific frequencies (measured in kilohertz, kHz), and converts them into electrical signals. These signals are processed and stored as digital recordings by the recorder. A microphone's **sensitivity** is a measure for the amplification or attenuation of the acoustic signal. The **frequency response** shows its relative sensitivity (measured in decibels, dB) at different frequencies. Since frequency responses are rarely linear, a microphone's sensitivity varies across frequencies, meaning sounds of differing intensities are captured unevenly. For a bat call to be recorded, its sound pressure level at a given frequency must exceed the microphone's **internal noise** or sound pressure level of the background. The ratio between the amplitude of the signal and the acoustic background is described by the **signal‐to‐noise ratio**. It should be noted that the value of the measured sound pressure of the noise strongly depends on the frequency width or FFT (fast Fourier transform) size of the analysis. The acoustic characteristics differ between microphone models and can also vary among individual microphones of the same model series. To account for these variations, microphones should be **calibrated** to measure their specific frequency response, ensuring accurate detection of bat calls for each individual unit (Agranat 2014).

The recorder captures bat calls as digital recordings and requires appropriate configuration. The **sample rate** of the recorder (in kHz) must be at least twice the highest signal frequency, as per the Nyquist theorem, to avoid aliasing (Landau 1967). For acoustic bat monitoring, a sample rate of 384 kHz is commonly used, accommodating echolocation frequencies exceeding 150 kHz (Barataud 2020; Russ 2021). Data are typically stored in uncompressed WAV format, preserving signal integrity for analysis. A **high‐pass filter**, or critical frequency, can be applied to exclude low‐frequency noise, with 16 kHz often used to effectively remove low‐frequency noise while retaining bat calls. **Pre‐ and post‐trigger** frames affect data capture and storage; longer frames increase the chance of recording faint calls but require more storage.

Full‐spectrum recording has become the standard in modern acoustic surveys and monitoring. While older recording techniques such as zero‐crossing, heterodyne recording, and frequency division were used before the widespread adoption of full‐spectrum methods (Brigham et al. 2004), full‐spectrum recording offers superior performance by capturing a broader frequency range, allowing for more comprehensive detection of bat calls across species and environments. The time expansion method often used in the past can be regarded as a special case of full‐spectrum recording. However, time expansion is not suitable for continuous monitoring of bat activity.

Bat calls recorded in the field are commonly identified by using specialized software. These tools apply the Fast Fourier Transform (FFT) to the full‐spectrum recordings to extract relevant features for further analysis. As a result of the FFT analysis, the bat calls can be visualized as time‐frequency plots (spectrograms or sonograms) for either automated or manual analysis. Subsequently, various filters can be applied to isolate bat calls within a recording (Clement et al. 2014). Once the calls are located, features such as minimum and maximum frequency, peak frequency, and call duration are compared to annotated reference data, either automatically through statistical methods or manually using a classification key or lifelong experience (Russo and Voigt 2016; Rydell et al. 2017).

Bats use echolocation calls (for simplicity, they are henceforth called calls) for navigation, orientation, prey detection, and communication with conspecifics, resulting in a range of call shapes and frequencies. They employ differently shaped calls depending on their task and surroundings (Russo, Ancillotto, and Jones 2018; Barataud 2020; Russ 2021) and different species can employ similar calls when solving the same tasks (Jones and Holderied 2007). Broadly speaking, European bat species can be grouped into three ecological guilds: open space, edge space, and narrow space foragers. Open space foragers, like the noctule bat ( *Nyctalus noctula* ), use low‐frequency calls with long ranges, while narrow space foragers, such as Bechstein's bat ( *Myotis bechsteinii* ), rely on high‐frequency, short‐range calls. Edge space foragers, like the common pipistrelle ( *Pipistrellus pipistrellus* ), employ mid‐frequency calls with medium ranges (Denzinger and Schnitzler 2013).

Acoustic surveys and monitoring of bats present challenges due to variations in detectability based on call intensity, call frequency, and call overlap (Russo and Voigt 2016; Russo, Ancillotto, and Jones 2018). Bat calls vary in intensity and frequency, with low body mass species, such as *Pipistrellus pipistrellus* and *P. pygmaeus* , emitting lower‐intensity, higher‐frequency calls, and high body mass species, such as *Nyctalus noctula* and *N. leisleri* , emitting higher‐intensity, lower‐frequency calls (Holderied and Von Helversen 2003). Call intensity (or “loudness”), measured in dB SPL (sound pressure level), decreases with increasing range due to the geometrical spreading of sound waves and is further attenuated by atmospheric and environmental factors. Atmospheric attenuation is primarily influenced by temperature (°C), relative humidity (%), and air pressure (Pa), while sound wave scattering and absorption increase in cluttered environments. Thus, cluttered environments, such as forests, cause significantly greater attenuation than open areas like grasslands. Higher‐frequency calls are more susceptible to attenuation, which limits the detection range of forest‐dwelling bats that emit high‐frequency, low‐intensity calls, often to just a few meters. In contrast, bats in open environments produce lower‐frequency, higher‐intensity calls, which experience less attenuation and can be detected over distances exceeding 50 m (Stilz and Schnitzler 2012; Jakobsen et al. 2013; Russ 2021). Furthermore, some bat calls can be more readily identified to the species level due to distinct call features, whereas others lack such unique identifiers. In Europe, genera such as *Plecotus* and *Myotis* exhibit considerable overlap in call characteristics. To address this, bat calls that cannot be reliably assigned to a species are commonly grouped into “sonotypes,” representing calls identifiable only to a broader taxonomic or functional group. Consequently, when assessing bat populations or environmental impacts, the absence of a detected call does not necessarily indicate the absence of a species.

This is further exacerbated by the fact that bat activity in acoustic surveys or monitoring is an interpretation of the recorded data rather than a direct observation. Unlike mist‐netting, where captured individuals and species can be visually quantified, acoustic sampling can only provide an indirect estimate, influenced by how the recorded calls are interpreted (Carvalho et al. 2023). Various metrics, such as bat passes or activity minutes, are commonly used to quantify bat activity, but the chosen metric significantly influences the results. Different interpretations can yield similar outcomes, and methods strongly affect the final estimate of activity (Miller 2001). The agreement between different automatic identification software tools or experts can be quite low, to a point where the results differ significantly (Jennings et al. 2008; Armitage and Ober 2010; Rydell et al. 2017; Solick et al. 2024).

In the end, acoustic surveys and monitoring offer a non‐invasive and effective approach to study bats, but its utility depends on the standardization of methodologies and comparability of results. The diversity of parameters, ranging from hardware configurations (Adams et al. 2012) to call identification protocols (Rydell et al. 2017), introduces variability that complicates cross‐study comparisons. For local monitoring to contribute meaningfully to larger‐scale population assessments, data must be interpretable across the wide range of methods currently employed. While approaches to improving data comparability and reusability have been developed in other fields, such as through the FAIR principles (Wilkinson et al. 2016), the challenges faced by acoustic bat research are similarly tied to issues of data discoverability, sharing, and integration. These guidelines emphasize the need for standardized formats, rich metadata, and clear provenance to support reproducibility and collaboration across studies. This prompts a critical question we aim to address: How comparable are acoustic bat research studies in Europe, given the methodological diversity? To investigate this, we focused on studies conducted in forest habitats where bat genera such as *Myotis* and *Plecotus*, which produce high‐frequency, frequency‐modulated calls that can complicate acoustic identification, occur. Based on our research question, we formulated two hypotheses: First, we hypothesized that the choice of hardware and software, and the subsequent analysis influence the number of recorded calls and the derived bat activity. We predicted (1) a huge variety of hard‐ and software, with differences in recording setups, identification protocols, and activity metrics, which result in heterogenic data with varying temporal and taxonomic resolution, and (2) that employing different identification software further contributes to discrepancies in detection and classification across different studies. Second, we hypothesized that this methodological heterogeneity requires transparent reporting of applied methods to avoid limiting the comparability of studies. We predicted that incomplete reporting will be common in our reviewed studies, which will impede cross‐study comparability, and that interpreted or aggregated data cannot be reinterpreted at finer taxonomic or temporal resolution if access to the underlying data is lacking. Finally, we propose a framework that identifies the crucial parameters of acoustic bat monitoring and provides guidance on how to report them accurately.

## 2 Material and Methods

### 2.1 Literature Research

We adhered to the “Preferred Reporting Items for Systematic Reviews and Meta‐Analyses” (PRISMA) protocols (Page et al. 2021). We conducted a comprehensive search of the *Web of Science* database using the following search parameters: “Chiroptera” OR “Bats” AND “Wood” OR “Forest” OR “Activity” OR “Acoustic Monitoring”. Additionally, we used the advanced search feature of *Google Scholar* with the same search parameters to identify additional publications and included the 200 top ranking publications. All resulting publications published between the 1st of January 2008 and 1st of June 2024 were considered. This timeframe was selected due to significant advancements in acoustic monitoring technology around 2008, including full‐spectrum ultrasonic recorders and automated call identification tools, which enhanced data consistency and study comparability. We focused on forests to include forest‐dwelling bats, as outlined in our hypothesis. Searches were conducted in English.

### 2.2 Article Screening and Selection

All publications were reviewed by a single evaluator (J. A.) to maintain consistency. A standardized protocol was implemented to ensure uniform assessment of all publications, specifying explicit inclusion criteria. During the initial screening of publications, we examined both the title and abstract to exclude studies that did not focus on bat monitoring within forests or woodlands in Europe. Any ambiguous cases were retained for further consideration in the subsequent screening round, focusing specifically on the forest subset of the data. Next, we assessed the full text of the preselected studies to see if they met our specified inclusion criteria. To be included in our review, each study had to fulfill all four criteria:

1. The publication reported the results of an acoustic bat survey or monitoring using at least one ultrasonic recording device;
2. The publication focused on bat activity within a range of forested environments, including forests, forest edges, clearings, and forest roads or tracks;
3. The publication provided data on all recorded bat species, with no species being preemptively excluded due to the research focus;
4. The survey or monitoring was conducted in Europe.

We extracted information from each retained paper, investigating the recording setup (recording device and microphone; critical frequency or high‐pass filter in kHz; sample rate in kHz; pre‐ and post‐trigger in ms; microphone used; height of the microphone; monitoring period), the species identification method (manual or automatic call identification; manual vetting; identification software), the bat activity definition (e.g., bat passes or activity minutes, resolution of the data), and the availability of the generated data (within the Supporting Information or in repositories).

Data availability was categorized into four groups: (1) raw data, (2) annotated records, (3) activity summaries, and (4) model output. Raw data refers to unprocessed audio recordings. Annotated records include recording names, timestamps, and assigned species labels. Activity summaries represent interpreted data with applied bat activity definitions, while the underlying recordings are not accessible. Model output comprises results from ecological models, with no access to the original acoustic data.

### 2.3 Frequency Response Acquisition

We collected frequency response data for the microphones used with the respective recording devices. This data was obtained from manufacturers' websites or through direct email requests. The dataset is inconsistent, with microphone attenuation and gain described using varying units and reference standards, complicating direct comparisons. For clarity, we chose to display only the most commonly used microphones, excluding those appearing in two or fewer of the retained publications. Frequency response data were not collected for all microphones, as certain models were either outdated and discontinued or custom‐built, making the information unavailable.

The frequency range selected for analysis spans from 0 to 160 kHz. The lower limit corresponds to the echolocation calls of *Tadarida teniotis* , the species with the lowest frequency echolocation calls in Europe (between 8 and 14 kHz), while the upper limit encompasses the calls of *Myotis nattereri* , which emits the highest echolocation call frequency among European bat species (up to 150 kHz). This range also includes the low‐frequency social calls of European bat species, such as *Nyctalus noctula* , *Eptesicus nilssonii* , and *Vespertilio murinus* , which can fall below 10 kHz. This selection ensures that the frequencies of all relevant European bat species, including both echolocation and social calls, are adequately covered, facilitating comprehensive analysis of bat activity across a broad spectrum of species (Russ 2021).

### 2.4 Conceptual Dataset for Bat Activity Scenarios

We created a completely fictional conceptual dataset to examine the impact of varying bat activity definitions and highlight challenges in interpreting acoustic data. The dataset reflects three hypothetical scenarios mimicking plausible bat behaviors observed in European forests, based on our experience with bat activity in this habitat. The first represents bats emerging from roosts after sunset, following a distinct flight path to an initial destination, such as a feeding site or water source. The second simulates high‐intensity foraging activity, characterized by frequent feeding interspersed with periods of perching or short commutes. The third scenario depicts a low‐activity commuting corridor used by bats traveling between roosting, feeding, or drinking sites.

To improve interpretability, activity was binned by minutes and hours rather than being aggregated per night, as commonly done in studies. The dataset assumes consistent recording conditions, with identical devices and settings across all scenarios. Differences in recorded activity reflect behavioral variations rather than sampling or species‐specific factors.

## 3 Results

### 3.1 Parameters in the Literature

**FIGURE 1:**
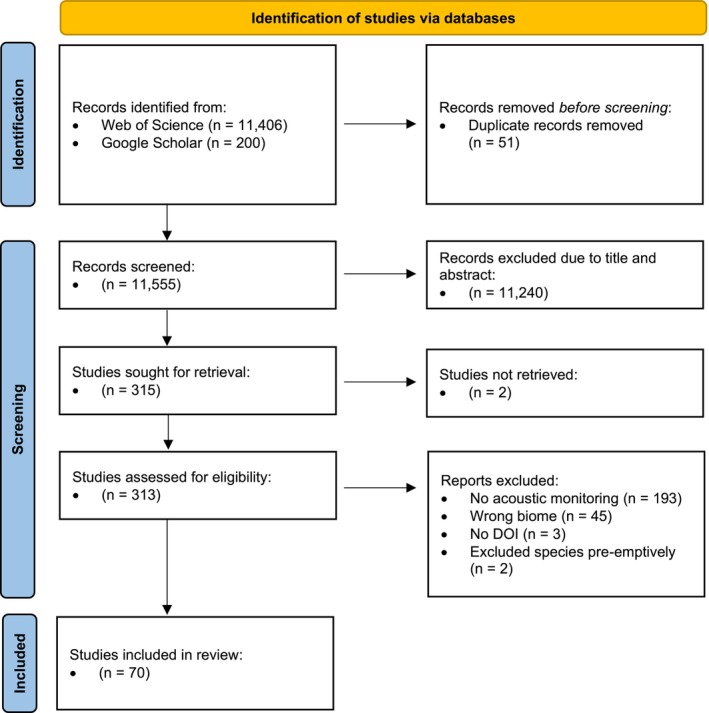
PRISMA flow diagram illustrating the review process for studies on acoustic bat monitoring in European forests based on the criteria: (1) The publication reported the results of acoustic bat monitoring using at least one ultrasonic recording device; (2) The study focused on bat monitoring within a range of forested environments, including forests, forest edges, clearings, and forest roads or tracks; (3) The publication provided data on all recorded bat species, with no species being preemptively excluded due to the research focus; (4) The monitoring was conducted in Europe.

Our initial screening identified 11,606 publications, with 11,406 sourced from Web of Science and an additional 200 from Google Scholar. We excluded 51 duplicates and rejected 11,240 studies based on their titles and abstracts, as they clearly did not address bats or acoustic monitoring, retaining 315 for further review. Two documents could not be retrieved and were excluded from the subsequent full‐text screening, during which we rejected 193 studies for not employing acoustic monitoring (but mainly mist netting and telemetry), 45 studies for focusing on incorrect habitats (primarily grassland), and two for focusing on a single species, thereby failing to meet all four of our inclusion criteria. Finally, three publications were excluded because they lacked DOIs, making it difficult to access the full text for review and data extraction. This process resulted in 70 retained publications, from which we extracted information relevant to our study on recording setup, species identification, and data interpretation (Figure 1).

**FIGURE 2:**
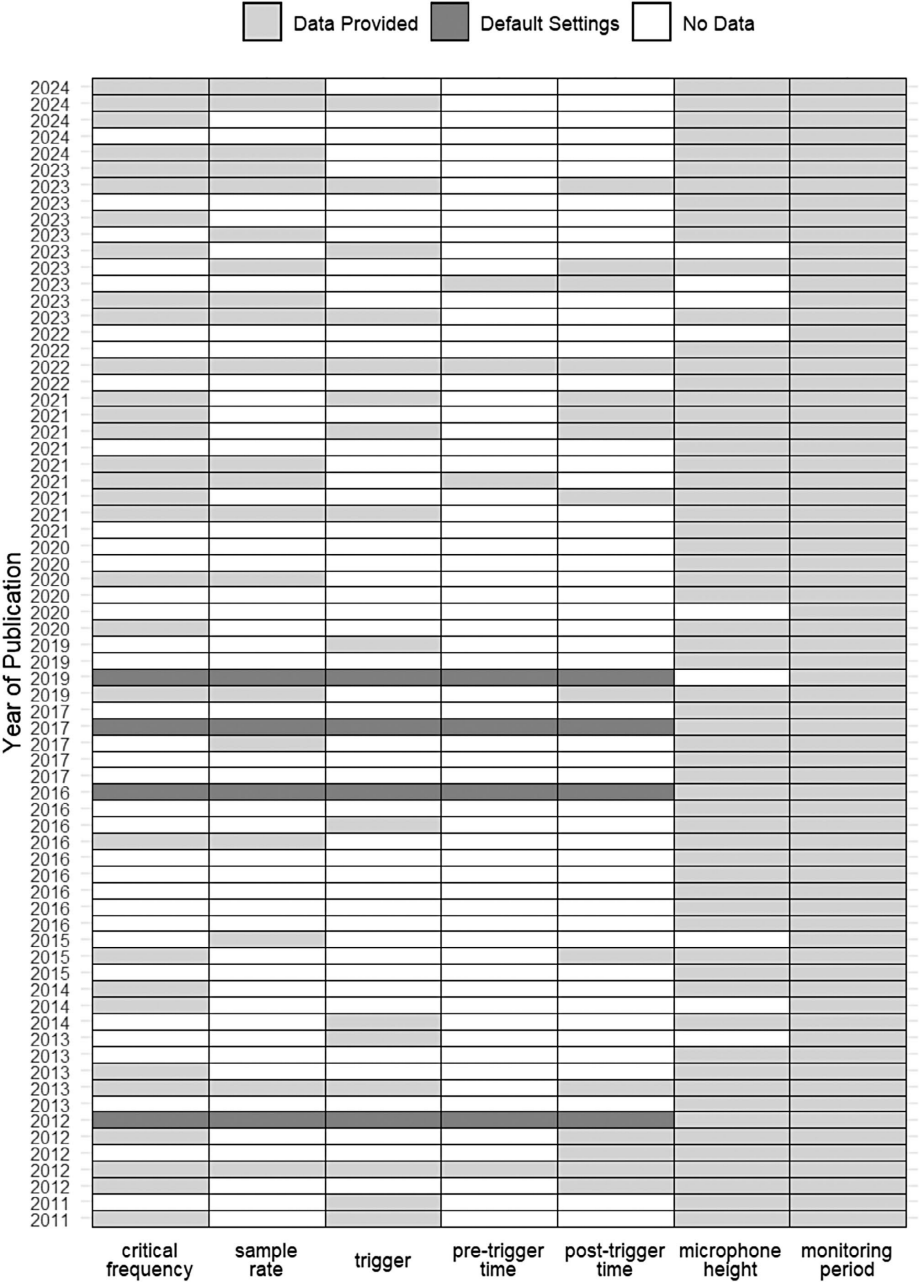
Summary of recorder and monitoring details reported in the reviewed publications. Each row represents an individual study, while the columns indicate the presence (light gray) or absence (white) of specific information (*x*‐axis). Dark gray signifies default settings, meaning the information was not explicitly stated, but the study applied the default settings provided by the equipment.

The review of the 70 retained publications revealed that reporting of basic recorder settings was often incomplete, with 91.5% of studies failing to provide full details. While all studies identified the recording devices used, only six offered comprehensive information on key parameters. Of these six, four referenced default values without providing complete details, requiring further consultation of the respective manuals. Specifically, 33 studies (47.1%) reported the high‐pass filter, or critical frequency; 24 (34.3%) provided the sample rate; 21 (30.0%) specified the trigger specifications (e. g. heterodyne, static, dynamic); 8 (11.4%) included pre‐trigger times; and 19 (27.1%) detailed post‐trigger times. All studies stated their monitoring period, and 61 (87.1%) mentioned microphone height. Over the study period, an increase in the reporting of sample rates and critical frequencies was observed, suggesting a more thorough documentation. However, other key recorder settings remained largely unreported (Figure 2).

### 3.2 Recorders and Microphones

**FIGURE 3:**
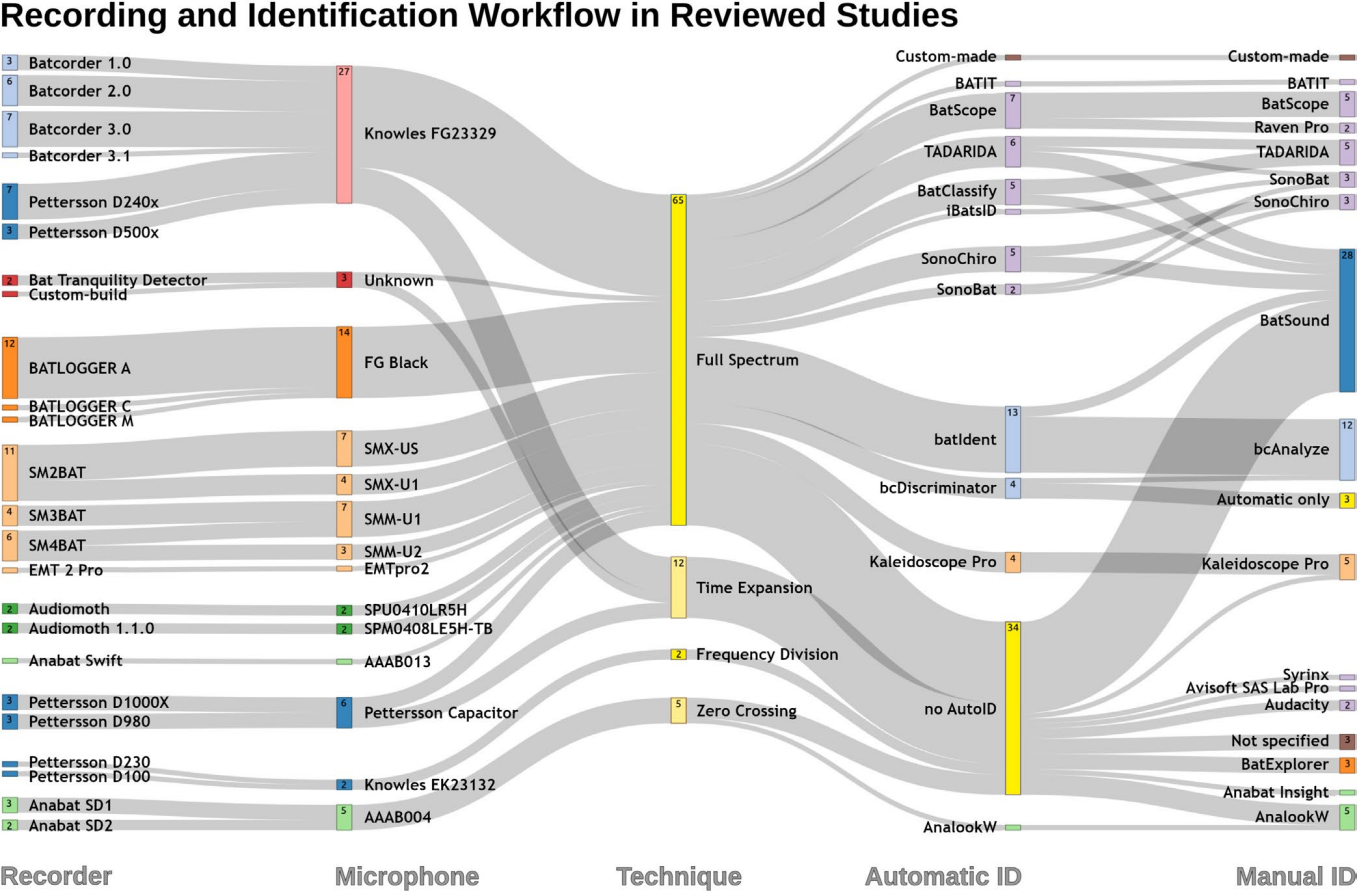
Overview of the recorders used in the reviewed studies, including their microphones, recording techniques, and the software applied for automatic identification and manual vetting. Colors are used to enhance readability and correspond to the respective manufacturer of the device or software. Each column represents the complete dataset, with proportions adding up to 100%, and absolute numbers (> 1) displayed inside the boxes. Note that some studies employed more than one recording device model.

Our review of 70 studies identified 26 distinct recording devices and 13 different microphones, as well as two unidentified microphone models. Figure 3 illustrates the various recorder‐microphone combinations and their respective recording techniques. The most frequently used recorder was the BATLOGGER A/A+, followed by the SM2BAT(+). Among the microphones, the Knowles FG23329 was the most common, featured in all Batcorder systems (ecoObs) and the Pettersson D240x and D500x models (Pettersson Electronic AB).

Full‐spectrum recording was the predominant technique, accounting for 78.6% of all studies, while time‐expansion and zero‐crossing recording devices were employed in 12.9% and 7.1% of the studies, respectively. One study still employed a frequency‐division recorder (1.4%). The latter techniques occurred predominantly in studies published before 2017, reflecting the shift toward full‐spectrum recording described earlier. All studies recorded bat calls eligible for subsequent analysis on computer systems.

### 3.3 Frequency Responses

**FIGURE 4:**
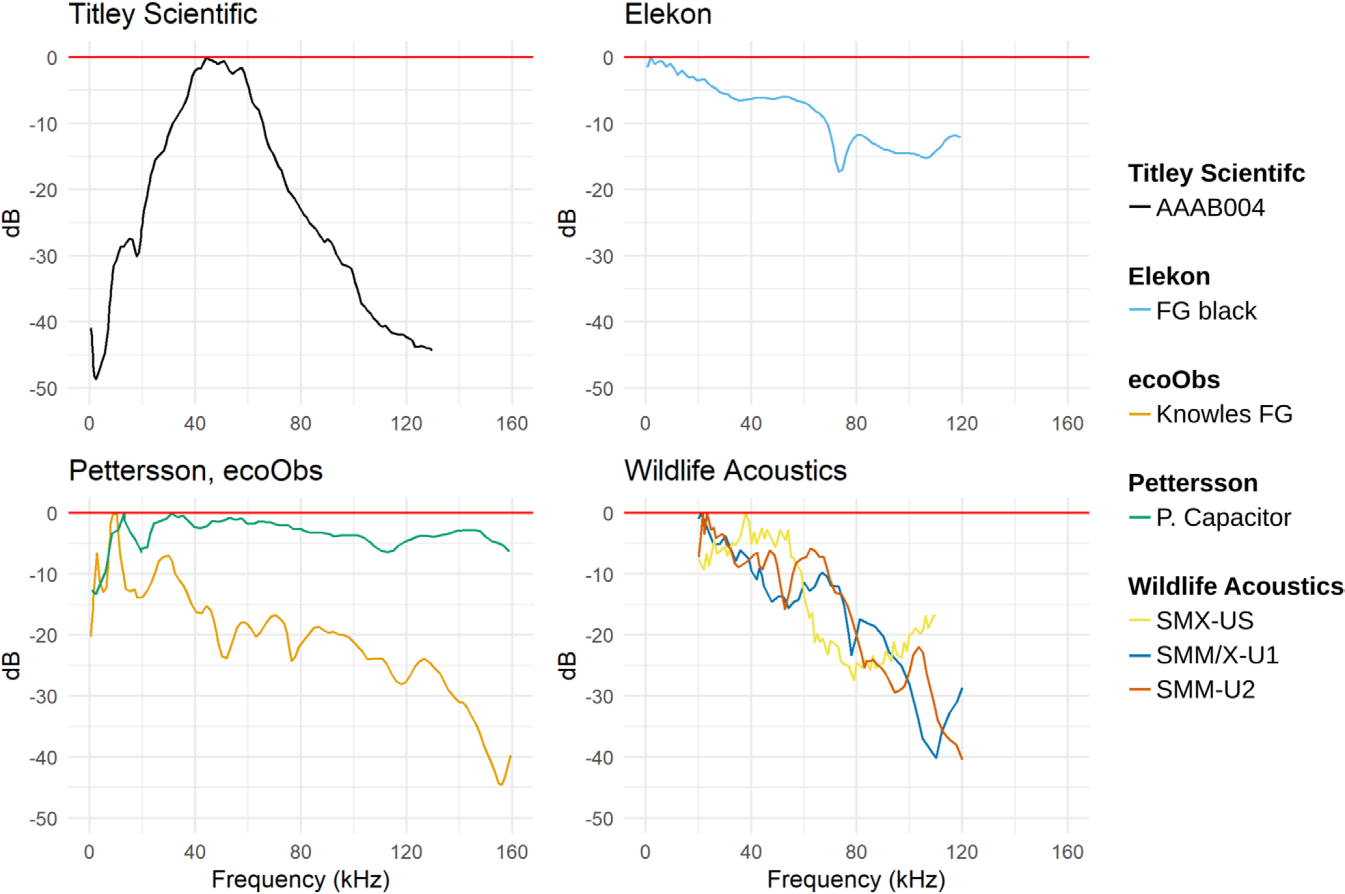
Frequency responses of the AAAB004 microphone used in the Anabat SD1 and Anabat SD2 recorders (Titley Scientific), the FG Black microphone used in various Elekon AG recorders, the Knowles FG 23329 microphone employed in all Batcorders (ecoObs GmbH), the Pettersson Capacitor microphone used in Pettersson Elektronik AB devices, and the SMX‐US, SMX‐U1, SMM‐U1, and SMM‐U2 microphones by Wildlife Acoustics. The intersection of the microphone‐specific graphs and the red line at 0 dB represents the frequency of highest sensitivity for each microphone. Sensitivity decreases with increasing deviation from 0 dB, reducing the likelihood of detecting signals at the given frequency. A flat frequency response indicates consistent amplification or attenuation across frequencies, reflecting good microphone performance.

We identified 13 distinct microphone models across the studies, along with two unidentified models: a custom‐built microphone reported in one study and microphones used in the bat detector model Tranquility (D. Bale, UK; discontinued) in two studies. Eight microphones were used in more than two studies. The frequency response curves for these microphones demonstrate substantial variability in sensitivity across different frequency ranges from 0 to 160 kHz (Figure 4). The frequency response displays the relative sensitivity of a microphone across a range of frequencies. Microphone sensitivity describes how acoustic signals at different frequencies are converted into electrical signals. Over the frequency range, the relative sensitivity can vary, meaning the microphone's electrical output may not match the signal intensity exactly, resulting in amplification or attenuation.

As signal intensity is a key factor in determining the detection range of recording setups, a microphone's frequency response significantly influences the maximum range at which bat calls can be recorded. Since every microphone exhibits a relative sensitivity, some degree of amplification or attenuation is always present. A microphone with a flat frequency response across the frequency range will have uniform amplification or attenuation, ensuring accurate recording of bat calls across frequencies. In contrast, a microphone with varying sensitivity across frequencies will result in less accurate recordings at certain frequencies. Additionally, even microphones of the same model can exhibit different relative sensitivities, which can only be assessed when the microphone is calibrated to account for its specific bias. To ensure comparability of recorded data, it is essential to account for the individual sensitivity of each microphone. Variations in sensitivity across microphones can lead to discrepancies in the amplitude of bat calls, which can subsequently affect the accuracy of analyses and interpretations of the results.

The most commonly used microphones in our reviewed studies vary considerably in their frequency responses (Figure 4). The AAAB004 microphone, employed in the Anabat SD1 and SD2 recorders (Titley Scientific), exhibits a peak sensitivity around 40–50 kHz, with a subsequent decline extending toward 130 kHz (D. Thompson, Titley Scientific, personal communication, 12.08.2024). The FG Black microphone, integrated into Elekon AG recording devices, demonstrates a more gradual decline in sensitivity across the frequency range, with a distinct dip at approximately 75 kHz (batlogger.com 2025). The Pettersson Capacitor microphone, used in the Pettersson D980 and D1000x recorders, maintains a largely flat frequency response, with minor attenuation observed at approximately 15 and 110 kHz. The Knowles FG23329 microphone, employed in the Pettersson D240x and D500x models, as well as in all Batcorders (ecoObs GmbH, versions 1.0, 2.0, 3.0, and 3.1), shows a gradual decline in sensitivity with increasing frequency, accompanied by minor fluctuations (L. Pettersson, personal communication, 09.08.2024; V. Runkel, personal communication, 01.07.2024). Wildlife Acoustics uses microphones incorporating Knowles sensors in their Song Meter 2–4 Bat recording systems, with MEMS sensors used in the SMX‐US model and FG sensors in newer versions. The SMX‐US microphone presents a flat frequency response between 20 and 55 kHz, followed by a stepwise decline, with a relatively flat region in the higher frequencies. The SMM‐U1 and SMX‐U1 microphones share an identical frequency response (Wildlife Acoustics 2018), characterized by a flat response at lower frequencies and a gradual decline toward higher frequencies, a pattern also observed in the SMM‐U2 model (Wildlife Acoustics 2025).

Ultimately, the choice of microphone influences the maximum range at which bat calls can be detected. Determining the true detection ranges of individual microphones is complex and beyond the scope of this paper (see Stilz and Schnitzler 2012). For example, comparing the frequency responses of the Titley Scientific AAAB004 and the Elekon FG Black, we expect the common noctule, with frequencies around 18–19 kHz, to be more prominently detected in recordings made with the Elekon FG Black than with the Titley microphone, due to the significantly lower sensitivity of the AAAB004 in the lower frequency range. Conversely, pipistrelle bats, with frequencies around 45 kHz, should be detected more effectively by the Titley microphone.

### 3.4 Species Identification

**FIGURE 5:**
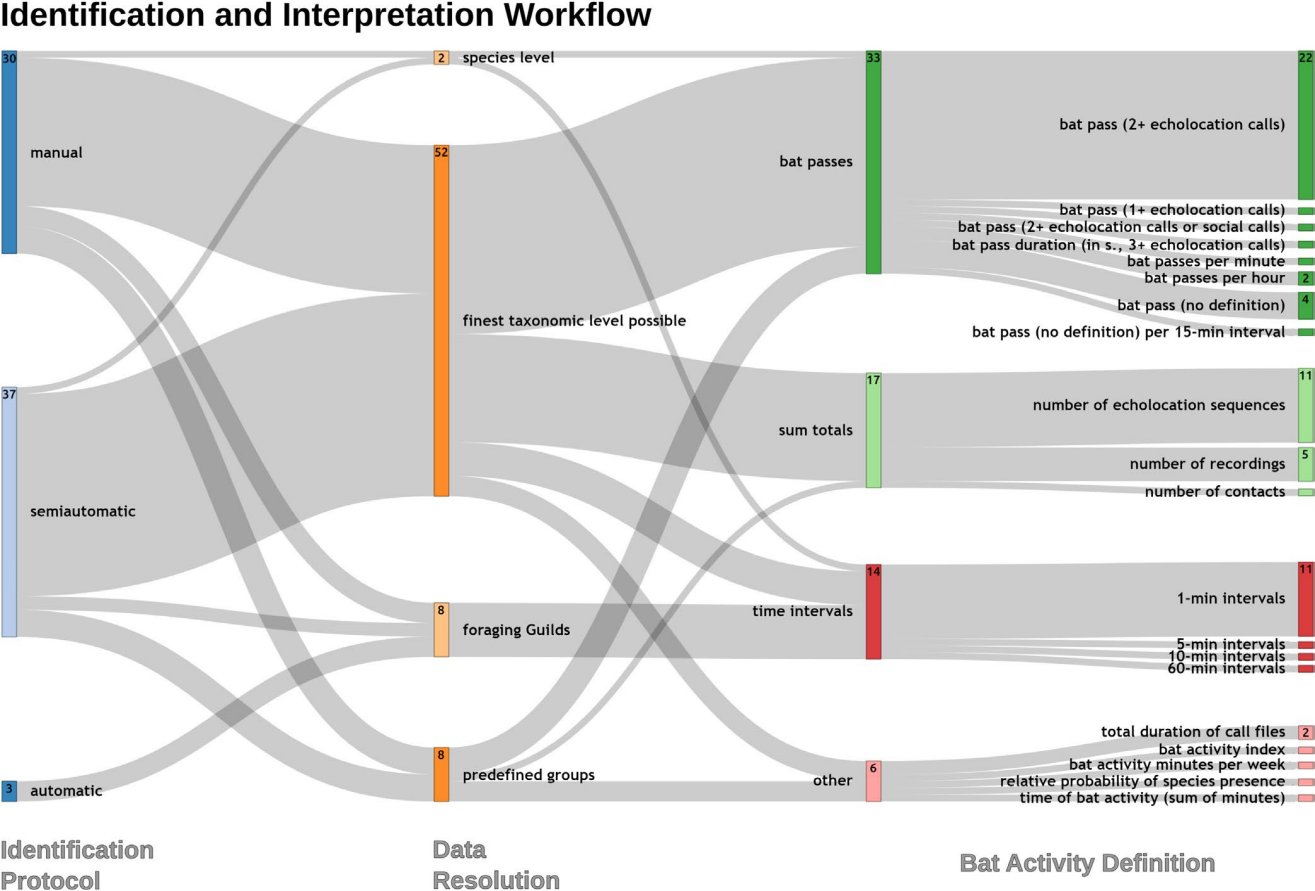
Overview of the identification methods used in the 70 reviewed studies, their data resolution, and the definitions of bat activity employed. Each column represents the complete dataset, with proportions adding up to 100%, and absolute numbers (> 1) displayed inside the boxes. Colors are used to enhance readability. Semiautomatic means that an automatic identification with manual vetting was conducted.

We identified three methods of bat species identification across the studies: manual, automatic, and automated identification with manual vetting. Of the 70 studies, 30 (43%) relied solely on manual identification, three (4%) used only automatic methods, and 37 (53%) employed automated identification with manual vetting. Twenty software solutions were used for species identification. Of these, eight were employed for both automatic identification and manual review, four were exclusively used for automatic identification, and eight were dedicated solely to manual review. One software package used for manual review was not specified. In total, 29 unique combinations of these 20 software solutions were documented, reflecting the variety of configurations applied to automatic and manual identification (Figure 5). Three publications (4%) stated the parameters they used for their Fast Fourier Transform (FFT), namely window type, overlap, FFT length, and frame size. Four additional publications stated only the window type, FFT length, and frame size, and one only the FFT length and frame size. The remaining 62 publications (89%) did not report any information on the applied FFT.

The lack of parameter reporting is problematic because the FFT parameters directly influence the accuracy of signal interpretation. There is an inherent trade‐off between frequency and time resolution. For example, with a sample rate of 384 kHz, a frame size of 1024 provides a frequency resolution of 375 Hz per interval or “bin”. While this allows for precise frequency analysis, it reduces the time resolution, meaning rapid changes in the signal may not be clearly captured. Conversely, a frame size of 512 improves time resolution (1.33 ms), but sacrifices frequency resolution, reducing the clarity of frequency details. The lack of clear reporting on these parameters makes it impossible to assess how studies prioritize either time or frequency accuracy, complicating comparisons across research.

Taxonomic resolution varied across the studies. In 37 publications, the *Myotis* genus was pre‐emptively grouped without further attempts at species‐level differentiation. In contrast, 33 studies attempted to identify *Myotis* to the species level. However, due to the inherent difficulty in distinguishing *Myotis* species from acoustic data, the majority of these calls were ultimately classified as the *Myotis* sonotype anyways. Aside from the *Myotis* genus, 52 studies aimed to categorize bat calls at the finest taxonomic level possible. In cases where species‐level identification was ambiguous, calls were assigned to broader sonotypes, such as Nyctaloid, Pipistrelloid, or *Plecotus*. Eight studies grouped bats by foraging guilds, while another eight employed predefined groups for classification. Only two studies restricted their analyses to species‐level identification, discarding any calls that could not be confidently identified.

### 3.5 Bat Activity Definition

Overall, we identified 20 distinct methodologies for translating the recorded audio files into measures of bat activity. These methodologies were categorized into four primary groups: bat passes, sum totals, time intervals, and “other.” A bat pass is defined as a sequence of echolocation calls produced by a single bat within a sound recording. It is counted when a predefined threshold of echolocation calls is met. Within our data, most studies used a threshold of two or more consecutive calls. The sum totals category includes bat activity quantified simply as the total number of recordings or echolocation calls, without further interpretation. Time intervals capture activity based on specific time frames, such as minutes. For example, when activity minutes are used, a minute is considered active if one or more calls are recorded, regardless of the number of calls. The “other” category includes alternative definitions of bat activity that do not fit within the primary groups and are less commonly used, such as total number of call files or bat activity minutes per week. Acoustic survey and monitoring data do not directly represent actual bat activity, as detection probabilities are influenced by factors such as species‐specific maximum recording distances and variations in bat behavior within the microphone's detection range (Adams et al. 2012; Russ 2021). For example, a bat may circle the microphone and produce multiple recordings, whereas another may pass briefly, generating only one or two detections (Miller 2001). Additionally, the absence of detections does not equate to an absence of bats.

**FIGURE 6:**
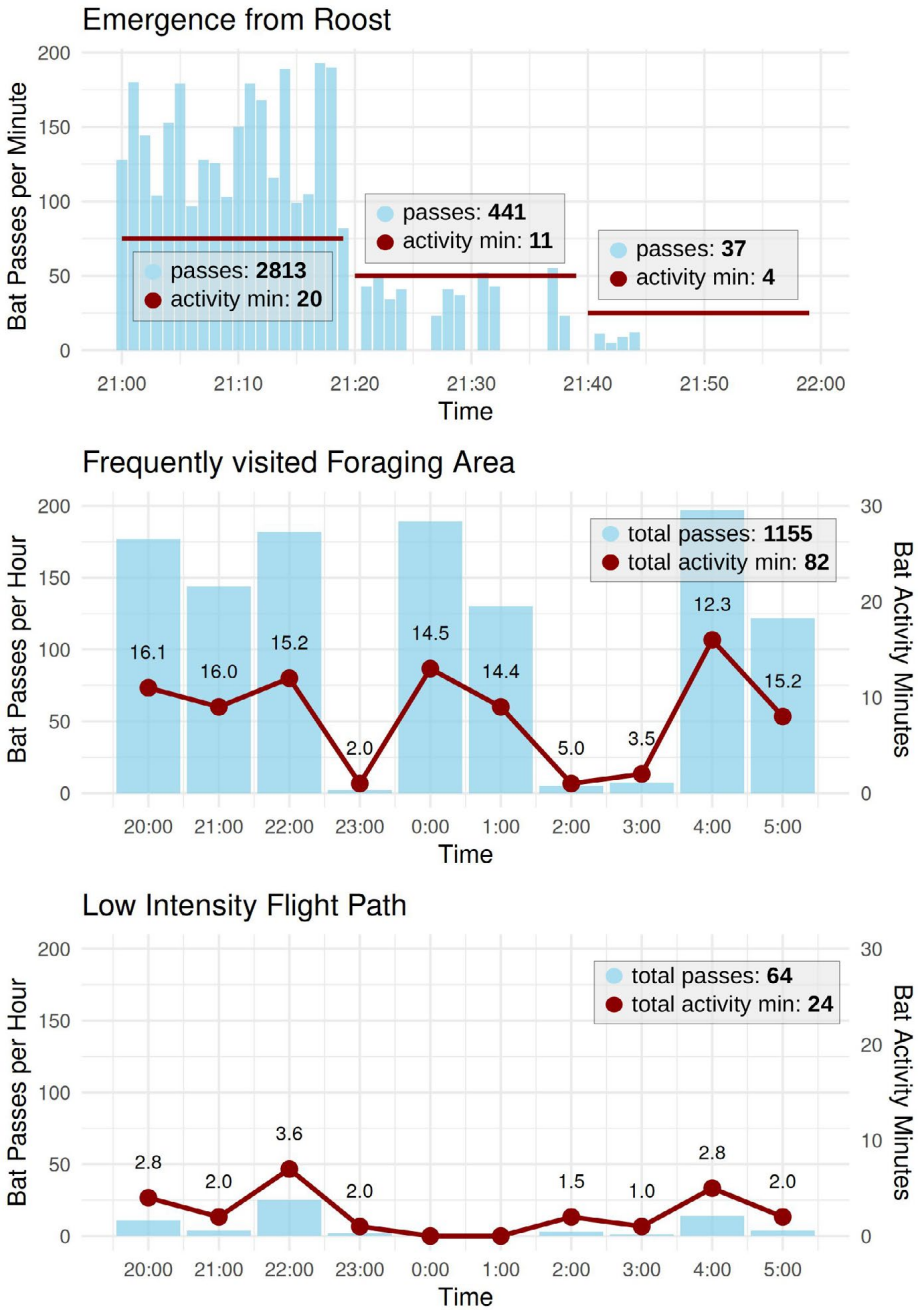
Three scenarios of acoustic bat monitoring highlight variations in data presentation. Gray boxes summarize typical ecological study outputs, while the graphs provide finer temporal resolution, aggregating bat passes (blue) and activity minutes (red) per minute or hour. The scenarios represent: (1) a flight path between a roost and a drinking site with a burst of early activity after emergence, (2) a high‐intensity foraging site with frequent but brief feeding events, and (3) a low‐intensity flight path where occasional bat passes result in more total activity minutes. These examples demonstrate that although bat passes and activity minutes are related, their ratio can vary substantially depending on the context.

Our conceptual dataset demonstrates the challenges in comparing different bat activity definitions by presenting three scenarios: roost emergence, a frequently visited foraging area, and a low‐intensity flight path. In the roost emergence scenario, high activity occurs during the first hour after sunset, with 2813 bat passes recorded in the initial 20 min, corresponding to 20 activity minutes. Activity decreases thereafter, with 411 passes corresponding to 11 activity minutes in the next 20 min and finally 37 passes corresponding to 4 activity minutes in the last 20 min. The ratio of bat passes to activity minutes declines over these intervals, from 140.7 in the first third to 40.1 and 9.3 in the second and final thirds, respectively. The foraging area scenario represents a site within a forest, such as a clearing or waterbody, where bats forage intensively but briefly. For the night, 1155 bat passes and 82 activity minutes were recorded, yielding an average ratio of 14.1 bat passes per activity minute. However, finer‐resolution data reveal ratios ranging from 2.0 to 16.1, depending on activity bursts. In the flight path scenario, a low‐intensity commuting corridor is modeled. Over the night, 64 bat passes and 24 activity minutes were recorded, with ratios ranging from 1.0 to 3.6. Activity is evenly distributed, reflecting sporadic visits by individual bats commuting between habitats (Figure 6).

These scenarios highlight that while bat passes and activity minutes are correlated, the relationship lacks consistency across contexts. The resolution of the data strongly influences interpretations, as aggregating data across an entire night can obscure critical details such as concentrated flight activity after roost emergence, short but intense feeding bouts, or low‐intensity commuting behavior. This variability highlights the difficulty in directly comparing bat activity metrics across different studies or contexts. Effective comparisons require access to the underlying data, allowing researchers to select the appropriate temporal resolution and bat activity definition for their specific research objectives.

### 3.6 Data Availability

The majority of our reviewed studies (73%) reported activity summaries, which included interpreted data based on bat activity definitions, without providing access to the raw recordings. In 25% of studies, data consisted solely of model outputs, which incorporated acoustic data but did not make the underlying recordings accessible. Interpreted data or model outputs cannot be used to reconstruct the raw data or annotated records, preventing reinterpretation or reproduction of the results. Raw data and annotated records were reported in one of our reviewed studies each.

### 3.7 Data Reporting Framework for Transparency in Acoustic Monitoring

We compiled a list of essential parameters to improve comparability and transparency in acoustic bat monitoring (Table 1). Each parameter should be explicitly addressed within a study, even if specific details are unavailable. For instance, if the frequency response cannot be provided, citing the microphone model and including its manual as Supporting Information would significantly enhance accessibility for readers. Additionally, acknowledging the absence of information on certain parameters can also be valuable. If, for example, the microphone was not calibrated, this should be clearly stated.

**TABLE 1:** Framework of key parameters for data collection, subsequent analysis, and result presentation in acoustic bat monitoring, essential for proper interpretation and reproducibility.

	Description
**Recording device**	
Microphone name and model	Specify the employed microphone model
*Frequency response*	Provide frequency response or link to manual
*Last calibration and deviation*	Date of last calibration and any deviation (in dB)
Recorder name and model	Specify the employed recorder model
*Firmware version*	Which firmware version was used during monitoring?
*Recording technique*	Were the recordings made in full‐spectrum or any other recording technique?
Recorder settings	Specify the settings of the employed recorder
*Sample rate*	For example, 256, 384, 512 kHz
*High‐pass filter*	The minimum frequency (in kHz) at which signals will be recorded
*Trigger activation*	Is there a trigger, if so, does the recording trigger automatically (static, dynamic) or manually?
*Pre‐trigger time*	How long is the recording time (in ms) before the trigger?
*Post‐trigger time*	How long is the recording time (in ms) after the trigger?
**Identification of bat calls**	
Identification Method	Specify how your recordings were identified
*Manual*	All bat calls were identified exclusively by experts
*Automatic*	All bat calls were identified only by auto‐identification tools
*Semi‐automatic*	All bat calls, or a subset, were identified using auto‐identification tools, and subsequently vetted manually
Programs used	Specify which software was used to analyze the recordings
*Manual identification*	Which software was used for manual identification?
*Automatic identification*	Which software was used for automatic identification?
Parameters of the FFT	Specify the applied Fast Fourier Transform parameters
*Window type*	For example, Hamming, Hanning or Rectangular
*Overlap*	Window overlap in %
*FFT length*	Data points used for FFT calculations, e.g., 256, 512 or 1024
*Frame size*	Number of samples per calculation, e.g., 512 or 1024
**Monitoring**	
Microphone height	Height of microphone placement (in cm or m)
Microphone orientation	Microphone orientation in degrees (°), can be vertical or horizontal
Monitoring period	When did the monitoring take place?
*Recording dates*	Start and end dates of the monitoring (Day, Month, Year).
*Recording sessions*	Nightly recording times relative to sunset/sunrise (hh:mm)
**Interpretation of data**	
Bat activity definition	Describe the method used to translate acoustic data into an activity index. Specify the metrics applied, such as bat passes, time intervals, or other measures.
Resolution	Specify how bat calls that could not be identified at the species level were handled. Were they excluded from the analysis, or grouped into sonotypes?
Sonotypes	Specify the sonotypes used in the study and provide details on which species are included in each group
Feeding buzzes	Specify whether feeding buzzes were identified in the data and if they were included in the study
Social calls	Specify whether social calls were identified in the data and if they were included in the study
Weight arguments	Specify if weight arguments were used to balance the varying recording probabilities of different bat species
Availability of recorded data	Specify whether the data is shared with others, available upon request, or stored in a repository
Accessibility of recorded data	Specify whether raw data, identified data with time and date stamps, or your interpretation of the acoustic data is provided

## 4 Discussion

Using the PRISMA protocols, we conducted a comprehensive literature research on acoustic bat monitoring in European forests. We screened 11,606 publications and selected 70 of those studies based on four inclusion criteria. As predicted, we identified a huge variety of recording and identification setups, with 60 unique combinations of recording devices, bat identification methods (manual or automatic), identification software, and definitions of bat activity across the retained studies. When accounting for the basic settings of the recording setup, no study authored by different researchers employed the exact same configuration. Overall, we found 26 different recording devices, 15 different microphones, 20 different bat call analysis software solutions, and 23 bat activity definitions with varying data resolution.

### 4.1 Recorders, Settings, Microphones

We identified a broad range of recording devices and microphones used in acoustic bat research over the past 15 years. This diversity reflects variations in institutional resources, research priorities, and budgetary constraints, as studies typically rely on equipment that matches available resources and specific objectives. Given the limited availability and high cost of specialized equipment, mandating the use of a single type of recorder or microphone is impractical. This applies to recorder settings as well: adjustments to sampling rates, trigger windows, and other parameters may suit one study but be irrelevant for another. For example, long‐term monitoring may require different methods than multi‐plot ecological studies (Jung et al. 2012; Stahlschmidt and Brühl 2012; Behr et al. 2023).

As hypothesized, variations in recorder settings can significantly affect results, highlighting the need for clear documentation of each parameter. Both default settings and custom adjustments should be specified, as these details are crucial for future data interpretation, particularly when device‐specific information becomes inaccessible due to discontinued models or company closures. For example, specifying the sample rate is critical to ensure accurate recording of high‐frequency bat calls and avoid aliasing, while the critical frequency is important for capturing lower‐frequency calls, such as social calls, which might otherwise be missed. Pre‐ and post‐trigger times also influence data interpretation: a short post‐trigger value may cause short‐interval echolocation calls, like those of *Pipistrellus pipistrellus* , to be grouped together, while longer‐interval calls, such as those of *Nyctalus noctula* , may be split. Thus, comparability in acoustic bat monitoring data should not rely on rigidly defined equipment or settings, but on precise documentation and transparency in methodology.

However, the capabilities of bat recorders differ significantly due to several factors: the frequency range and amplitude of bat calls, the increasing distance between the vocalizing bat and the recording device (Kunberger and Long 2023; Goodwin et al. 2024), and the acoustic environment itself. The cluttered environment of forests further attenuates call signals, with high‐frequency bat calls facing particular detection challenges due to the absorption of their sound by vegetation and other environmental obstacles (Stilz and Schnitzler 2012). Additionally, bat calls are not emitted with uniform intensity. Lighter bats generally produce calls of lower intensity compared to heavier bats, and call intensity can differ within the same species depending on environmental conditions and behavioral context (Schnitzler and Kalko 2001; Holderied and Von Helversen 2003). Thus, the distance a bat call can travel and be recorded depends on geometrical spreading, atmospheric and environmental attenuation, and the bat itself.

While studies have estimated detection ranges for bat calls, accurately quantifying these distances remains challenging due to the numerous factors influencing call propagation and recording conditions, as well as the microphone itself (Adams et al. 2012; Stilz and Schnitzler 2012; Russ 2021). The observed differences in microphone frequency responses within our data highlight a key factor impacting the comparability of acoustic bat monitoring data across studies. As such, specifying the microphone model and frequency response is essential for accurate interpretation, as variations in sensitivity affect the detectability of all bat species differently, especially in high‐attenuation habitats like forests. Routine microphone calibrations before and after fieldwork help to identify and quantify device‐specific sensitivity variations, allowing for the accounting of individual detection biases and improving data precision. Further, documenting placement parameters, including microphone height, will aid in assessing the potential influence of setup choices on recorded bat diversity (Müller et al. 2013). Establishing standardized reporting of these details will enable better replication and cross‐study comparisons, thus enhancing the transparency of acoustic monitoring findings, which is in line with our hypotheses and predictions.

We will briefly address two additional factors influencing the recording and interpretation of bat calls. First, the angle at which a bat calls relative to the microphone affects detectability, with the highest recording probability when the bat faces the microphone directly (Adams et al. 2012). This variation, known as the directional response, differs across microphones. Since bats inevitably approach microphones from various angles in the field, accounting for the directional response is crucial for interpreting detection probabilities and further highlights the need to specify recording hardware, particularly the microphone. Specifying the microphone model can therefore help contextualize detection rates and support meaningful interpretation of acoustic data.

Second, detection probability varies due to differences in call structure, frequency, and intensity. Long, quasi‐constant low‐frequency calls are more likely to be recorded than short frequency‐modulated calls, due to their increased maximum detection distance. To account for this variation, one reviewed study applied a detectability coefficient. In this study, the authors adjusted the number of call sequences by a factor based on detection probability. For example, calls from *Nyctalus noctula* (long, quasi‐constant low‐frequency calls), detectable up to 80 m, were multiplied by a factor of 0.25, while calls from smaller *Myotis* species (short frequency‐modulated calls), often only detectable up to a few meters, were multiplied by 1.67 (Ciechanowski et al. 2024). While such adjustments may improve comparability across species within a study, we emphasize the importance of also providing unweighted data to support reinterpretation and cross‐study comparison of the same bat species.

### 4.2 Identification—Manual or Automatic, FFT, Resolution

All reviewed publications recorded bat calls in either full‐spectrum (78.6%), time‐expansion (12.9%), zero‐crossing (7.1%) or frequency‐division (1.4%). These recordings can be analyzed using spectral analysis to generate time‐frequency plots. However, comparing data across different recording methods is challenging due to differences in signal representation. Zero‐crossing and frequency‐division techniques provide real‐time playback, limiting the capture of detailed spectral information, particularly harmonics and amplitude. Time‐expansion preserves the full waveform but requires scaling adjustments for accurate frequency and time interpretation. Full‐spectrum recording captures the entire ultrasonic waveform, including harmonics and frequency sweeps, providing the most comprehensive signal representation. These variations in resolution, frequency accuracy, and detail make direct comparison difficult and require extensive knowledge of signal processing (Brigham et al. 2004; Zamora‐Gutierrez et al. 2021). Significant advancements in recording technology have established full‐spectrum recording as the standard technique for all current‐generation bat recording devices. Our following discussion is therefore based on the use of this recording method.

The Fast Fourier Transform (FFT) is a widely used method for generating spectrograms in acoustic studies. While FFT is a standardized algorithm, the choice of software mainly affects the user interface and customization options. To ensure reproducibility and consistency in data interpretation, key parameters, such as FFT length, frame size, window type, and overlap, should be explicitly reported, as these parameters define the trade‐off between time and frequency resolution in signal analysis. In our review, only three of 70 publications provided these settings, which may introduce variability in call classification and analysis, complicating replication. Reporting these parameters would improve transparency and comparability across studies (Bradbury and Vehrencamp 1998; Brigham et al. 2004).

The resulting FFT spectrograms can then be analyzed manually or automatically. We identified 20 different software solutions for acoustic data analysis within our data, of which eight were employed for both automatic identification and manual review, four were exclusively used for automatic identification, and eight were dedicated solely to manual review.

The variety of manual identification software does not affect data comparability, as software choice primarily influences graphical interfaces and customization options. Signal analysis differences primarily arise from FFT settings, which remain consistent across software due to its standardized algorithm. Nevertheless, manual identification is labor‐intensive, requires experience, and is inherently subjective. Classification accuracy depends on the classifier's expertise, and species with overlapping call types, like those in the genus *Myotis*, are frequently misclassified, even by experts with years of experience (Jennings et al. 2008; Rydell et al. 2017).

Automatic bat call identification accelerates workflow, reduces manual labor, and improves repeatability (Skowronski and Fenton 2008). We found 12 different dedicated automatic bat call identification software programs being used within our reviewed publications. All software follows the same initial approach, where bat calls within the recorded data are located. This is achieved by amplitude threshold filtering (ATF), detecting areas of smooth frequency change (ASFC) or detection of search criteria (SC) and cross‐correlation (CC) of signal spectrograms (Walters et al. 2013). Different identification software packages use different approaches, by employing either one or a combination of the methods described. While every approach has its unique benefits and challenges, these different approaches affect the number of calls detected within an acoustic dataset, with some software being more adept at capturing quieter calls or specific call structures, as we predicted (Clement et al. 2014).

Once a call is detected, defined features are extracted from each call, such as peak frequency, call length, start and end frequency, and subsequently compared to reference datasets to assign species labels. This feature extraction and classification is typically performed by machine learning models, such as hidden Markov models (HMM) or Gaussian mixture models (GMM) or ensemble learning techniques such as random forests (Zamora‐Gutierrez et al. 2021). Limited documentation on algorithm methods and reference databases provided by the authors of these auto‐identification tools adds complexity and uncertainty when interpreting results across studies employing different software (Walters et al. 2013).

Agreement levels between software vary, depending on the genera or species analyzed. For some European bat species, automatic feature extraction and identification perform with high precision when species‐specific call features are present. Examples include distinct frequency bands exceeding or falling below specific thresholds, as observed in several *Pipistrellus* species, or unique alternating call sequences characteristic of *Barbastella barbastellus* (Dietz et al. 2009; Skiba 2009; Barataud 2020; Russ 2021). Recordings with unique call features present allow for a high agreement between different software solutions (Rydell et al. 2017). However, if unique features are absent, additional parameters can be considered for species identification. For example, *Pipistrellus kuhlii* and *Pipistrellus nathusii* , as well as *Eptesicus serotinus* and *Eptesicus nilssoni* , emit calls whose frequencies overlap. Differentiation often depends on geographic distribution, highlighting the need to consider the study area for accurate species identification.

In addition to species‐specific challenges, software capabilities differ in their approach to analyzing bat calls. Kaleidoscope (Wildlife Acoustics) focuses exclusively on detecting echolocation calls, while batIdent (ecoObs) distinguishes between echolocation calls, feeding buzzes, and social calls. Social calls are particularly valuable for distinguishing species with overlapping echolocation frequencies, such as *Vespertilio murinus* , *Eptesicus serotinus* , and *Nyctalus leisleri* , while also providing insights into behaviors like foraging and social interactions (Dietz et al. 2009; Skiba 2009; Barataud 2020; Russ 2021). Nevertheless, many social calls cannot be reliably assigned to species level yet, as identification accuracy of classification tools depends largely on the quality and coverage of their respective training libraries. This corresponds to our prediction that different identification software packages increase discrepancies in detection and classification across studies.

Species with similar echolocation call structures, such as those in the *Myotis* and *Plecotus* genera or among genera with overlapping call frequencies, remain challenging for all bat identification software (Rydell et al. 2017). To address this, most reviewed studies adopt a semi‐automatic workflow: The automated identification is followed by manual vetting to minimize misclassification among species with similar echolocation characteristics, such as *Myotis* spp., *Plecotus* spp., and *Pipistrellus kuhlii* versus *P. nathusii* . When species‐level identification is unfeasible due to a lack of distinguishing features, such as species‐specific social vocalizations, or the sheer volume of data, classification often defaults to the genus level or to sonotypes. However, as noted earlier, manual vetting introduces subjectivity, even when guidelines are followed, reducing comparability with fully automated methods. In our dataset, over half of the reviewed studies (37 publications) grouped *Myotis* calls at the genus level, omitting individual species‐level information. Overall, 97% of studies categorized a proportion of their calls only on the genus level or as sonotypes, while only two publications excluded calls that could not be identified to the species level.

We cannot provide a definitive recommendation on the optimal identification method. However, we advocate for maximum transparency, including clear details on the software used, FFT parameters, classification keys applied, and the rationale for introducing specific sonotypes. Ideally, recorded data should be publicly accessible or available upon request to enable independent verification or alternative identification workflows. A standardized and precise automated identification system could enhance cross‐study comparability while reducing the workload for researchers. This approach could also help fill gaps in bat population data, which threaten European and global bat biodiversity (Frick et al. 2020). Solutions for automated bat identification could be achieved with the help of artificial intelligence. Jennings et al. (2008) demonstrated that automatic bat call identification using artificial neural networks performs as well as experienced manual reviewers. Other approaches using convolutional neural networks or transformer models could show a high precision of over 90% when identifying bat echolocation calls from species with overlapping call frequencies and similarly shaped calls, as for example within the *Myotis* genus. With the given dataset, these neural networks outperformed feature extraction software greatly (Rydell et al. 2017, Bellafkir et al. 2022, Schwab et al. 2023).

Recent developments in artificial neural networks show potential for improving automatic bat call identification, especially for distinguishing species where traditional feature‐extraction software underperforms. Neural networks can advance species‐level identification for all European bat species, with results that can be interpreted with a known level of error, ensuring comparability across studies. With sufficient training data, these networks can also identify social calls and feeding buzzes, providing additional insights into bat behavior and ecology (Vogelbacher et al. 2023). Accurate species‐level identification could enhance understanding of bat populations, which, although outside the scope of a study, would be valuable for future research. In the face of climate change, such advancements could help monitor population shifts. However, we believe that automatic bat call identification remains in the early stages of development, with a significant lack of annotated training data hindering further progress and no commercially available software, thus preventing broad application in diverse user groups.

### 4.3 Bat Activity Definition, Presentation of Data, Supporting Informations

Bat activity definitions strongly affect data interpretation. A single sound file can be classified as a bat pass, an activity minute, or a sum of echolocation calls, leading to discrepancies in large datasets. Factors such as recording length or pre‐ and post‐trigger times further complicate the relationship between the number of calls and recordings. As a result, the interpretation of acoustic data is heavily influenced by the chosen bat activity definition. Recordings of different bat activities, such as roost emergence, foraging events, and low‐intensity flight paths, cause the relationship between various activity indices to vary significantly (e.g., the ratio between passes and activity minutes). Furthermore, temporal patterns and the intensity of bat activity can be misrepresented or lost when data are aggregated, such as when summarizing results for a single night (Miller 2001; Adams 2013).

The underlying data of interpreted acoustic recordings, once analyzed with a specific bat activity definition, cannot be easily reconstructed for alternative interpretations or for altering temporal resolution. Therefore, as we predicted, cross‐study comparability and the reuse of acoustic data depend heavily on access to the underlying data. However, the majority of the reviewed studies (73%) presented data at an interpreted level, meaning they reported results after applying a bat activity definition. A smaller proportion (25%) used ecological models with bat call data as input but did not provide either the interpreted results or the underlying data, further limiting transparency. In contrast, only two studies provided data that allowed for reinterpretation: one shared the raw data, while the other provided annotated recordings with timestamps, enabling the application of a different bat activity definition.

While we do not advocate for a specific bat activity definition, as different research questions and experimental designs may benefit from varying definitions, we emphasize the need for transparent data presentation. The choice of definition may depend on the specific research goals or personal preference, but comparability across studies is crucial. Researchers should not only report bat activity results but also make the underlying data available, typically in the form of a spreadsheet containing all recorded data, annotations (either automatic or manual), and timestamps. This transparency would allow researchers to apply their own definitions, facilitating cross‐study comparability.

Although sharing raw data would be ideal, we recognize the substantial effort required to collect such data, which may make this impractical in some cases. Nevertheless, sharing raw data could benefit other research areas, such as ecological studies on bat vocal repertoires or broader population research (Gillam and McCracken 2007; Burchardt and Knörnschild 2020). Once a dataset is fully explored by the original researchers, it may hold potential value for others.

## 5 Conclusion

Our review highlights significant challenges in achieving comparability in acoustic bat research across Europe. As hypothesized, these challenges arise from substantial variation in recording devices, microphones, and identification protocols, and a general lack of transparency in reporting the respective methods. Most reviewed studies did not provide sufficient detail on equipment specifications, call identification protocols, or access to underlying data, hindering reinterpretation, limiting reproducibility, and cross‐study comparability. As a result, the majority of studies we reviewed could not be reproduced or reinterpreted. We emphasize that these issues are not a reflection of the competence or intent of the researchers involved. Instead, they highlight the need for a standardized approach to bioacoustic methodology. To contribute to this goal, we have defined a set of basic monitoring parameters that we believe should be consistently reported in acoustic bat studies (Table 1). Clear and standardized reporting of equipment parameters and settings would improve the interpretability and comparability of acoustic data across studies. Such documentation is also crucial for facilitating the replication of study designs, a cornerstone of scientific research, and would enable other researchers to validate and expand upon existing work. Increased collaboration within the bat research community could drive interdisciplinary research, enhance conservation efforts, and optimize the use of public funding.

## Author Contributions

**Jarno Asmus:** conceptualization (lead), data curation (lead), formal analysis (lead), investigation (lead), methodology (lead), visualization (lead), writing – original draft (lead), writing – review and editing (supporting). **Karl‐Heinz Frommolt:** funding acquisition (lead), project administration (lead), resources (lead), supervision (equal), writing – review and editing (equal). **Mirjam Knörnschild:** supervision (equal), writing – review and editing (equal).

## Conflicts of Interest

The authors declare no conflicts of interest.

## Supporting information


**Appendix S1:** ece371883‐sup‐0001‐AppendixS1.csv.

## Data Availability

The reviewed publications that form the basis of this study are available on Web of Science (https://www.webofscience.com) and are listed in the Supporting Information material. These data were obtained from publicly available resources: https://www.webofscience.com/.
